# Evaluation of Various Thiourea Derivatives as Reducing Agents in Two-Component Methacrylate-Based Materials

**DOI:** 10.3390/polym17152017

**Published:** 2025-07-23

**Authors:** Coralie Ohl, Estelle Thetiot, Laurence Charles, Yohann Catel, Pascal Fässler, Jacques Lalevée

**Affiliations:** 1 Université de Haute-Alsace, CNRS, IS2M UMR 7361, F-68100 Mulhouse, France; coralie.ohl@uha.fr (C.O.);; 2 Aix Marseille Université, CNRS, UMR 7273, ICR, F-13397 Marseille, France; laurence.charles@univ-amu.fr; 3 Ivoclar Vivadent AG, Bendererstrasse 2, FL-9494 Schaan, Liechtenstein

**Keywords:** redox initiators, redox agents, redox polymerization, thiourea, chemical mechanisms

## Abstract

Two-component dental materials are commonly used by the dentist for various applications (cementation of indirect restorations, filling of a cavity without layering, etc.). These materials are cured by redox polymerization. The (hydro)peroxide/thiourea/copper salt redox initiator system is well established and can be found in a wide range of commercially available dental materials. The thiourea is a key component of the initiator system. This study explores the influence of the nature of the thiourea reducing agent on the reactivity and efficiency of redox initiator systems. In this work, six different thiourea structures were investigated, in combination with copper(II) acetylacetonate and cumene hydroperoxide (CHP), to understand their impact on polymerization kinetics and mechanical properties of methacrylate-based materials. Various experimental techniques, including mass spectrometry (MS) and spectroscopic analyses, were employed to elucidate the underlying mechanisms governing these redox systems. The results highlight that thiourea plays a dual role, acting both as a reducing agent and as a ligand in copper complexes, affecting radical generation and polymerization efficiency. Structural modifications of thiourea significantly influence the initiation process, demonstrating that reactivity is governed by a combination of factors rather than a single property. Self-cure dental flowable composites exhibiting excellent flexural strength (>100 MPa) and modulus (>6000 MPa) were obtained using hexanoyl thiourea, N-benzoylthiourea, or 1-(pyridin-2-yl)thiourea as a reducing agent. The adjustment of the Cu(acac)_2_ enables to properly set the working time in the range of 100 to 200 s. These findings provide valuable insights into the design of the next generation of redox initiating systems for mild and safe polymerization conditions.

## 1. Introduction

**Figure 1:**
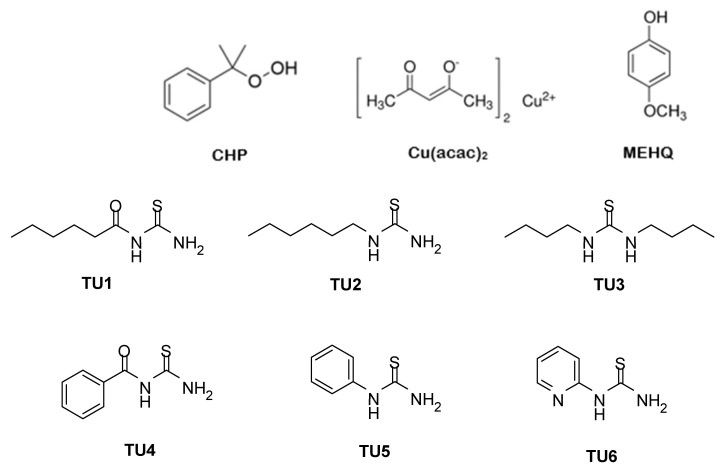
Structures of thioureas **TUs1-6**, **CHP**, **Cu**(**acac**)**_2_** and **MEHQ**.

Two-component dental materials, such as dual-cure luting composites, self-adhesive resin cements, or self-cure/dual-cure flowable composites, are broadly used in dental practices [1,2,3,4,5,6,7,8,9]. The main advantage of these materials in comparison to light-cure composites is their ability to cure in the absence of light. They are therefore particularly used for the cementation of indirect restorations. Indeed, after placement of the restoration, and depending on its thickness and opacity, only a limited amount of light can reach the uncured cement. A dark curing is consequently necessary in order to obtain efficient polymerization and high double-bond conversions. The use of dual-cure flowable composites to fill deep cavities is an additional interesting application field for two-component dental materials. This approach improves the workflow efficiency, as the dentist can save time by avoiding the application of several composite layers. Indeed, thanks to the dark curing, polymerization can also occur in deep and shadow areas that cannot be irradiated due to the material opacity. Two-component dental composites and cements are typically made up of an organic matrix combined with inorganic fillers (silanized glass fillers, spherical mixed oxides, ytterbium fluoride, etc.). The organic matrix mainly comprises a mixture of methacrylates, an initiator system, and stabilizers. Dual-cure materials are based on both a photoinitiator and a redox initiator system, whereas self-cure composites do not contain photoinitiators. The redox initiator systems consist of an oxidizing agent, a reducing agent and most of the time a catalytic amount of a metal complex. The oxidizing and reducing agents are incorporated into different components. The mixture of both components during the application triggers a reaction between the oxidizing and the reducing agents (which is often catalyzed by the presence of the metal complex), leading to the formation of radicals that are responsible for the polymerization of the dimethacrylate-based material. Peroxides, such as benzoyl peroxide (BPO), cumene hydroperoxide (CHP), and tert-amyl peroxide, are the most commonly used oxidizing agents in dental materials [10,11,12,13]. Hydroperoxides have been shown to be more attractive than BPO due to their improved stability. Indeed, although dental materials containing BPO must be stored in the refrigerator, formulations based on hydroperoxides are typically stable at room temperature. With regards to reducing agents, a wide range of compounds, including aromatic tertiary amines (in combination with BPO), ascorbic and barbituric acid derivatives, sulfinate salts, dihydropyridines, and thioureas has been evaluated in two-component dental materials [10,11,14,15,16,17,18,19,20,21,22,23,24,25]. The redox initiator system based on the combination of a hydroperoxide with thiourea is particularly attractive. Indeed, it exhibits an excellent storage stability due to the air insensitivity of thiourea derivatives. Copper salts are typically added as catalyst in order to tune the curing kinetics and the working time of the dental materials. Amongst the thiourea derivatives, acylthioureas have been mainly investigated [24,25]. The objective of this work is to evaluate the potential of various thioureas (by modifying the substitution of the nitrogen groups) as reducing agents in two-component dental materials. The compounds **TUs1-6** (Figure 1) were selected for this study.

In this contribution, the reactivity of **TUs1-6** in combination with CHP and a catalytic amount of Cu(acac)_2_ (different concentrations are considered) is firstly assessed in a methacrylate mixture. The reactivity is assessed by pyrometry, and the mechanical properties (flexural strength/modulus) of the cured materials are determined. The most efficient redox initiator systems are then evaluated in two-component dental composites. The flexural strength/modulus, double-bond conversion (DBC), and working time are measured. The influence of the nature of the thiourea structure on the efficiency of the redox reaction is discussed. Other redox initiating systems were also recently reported in other fields [26,27,28].

## 2. Experimental Section

### 2.1. Chemical Compounds

**Figure 2:**
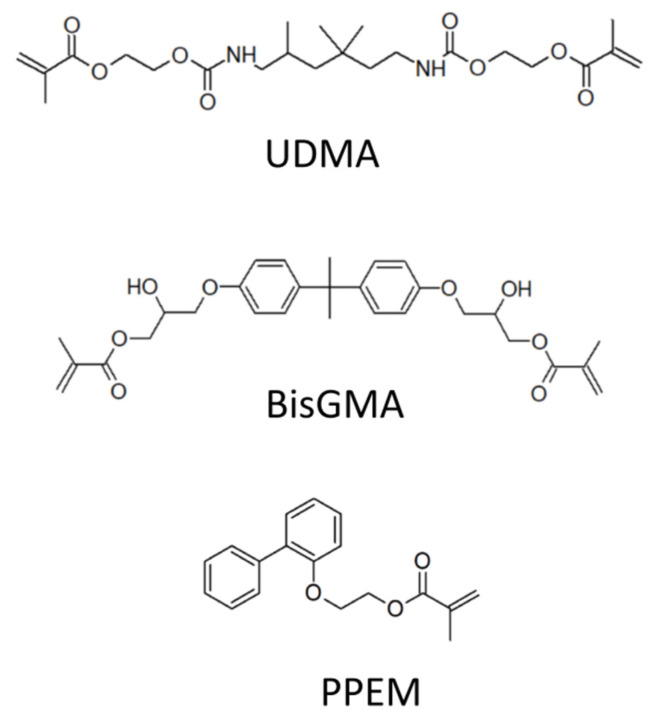
Structures of the Monomer Blend.

2,2-Bis-[4-(2-hydroxy-3-methacryloyloxypropoxy)phenyl]propane (Bis-GMA) was purchased from Evonik (Essen Germany). 1,6-Bis-[2-methacryloyloxyethoxycarbonylamino]-2,4,4-trimethylhexane (UDMA) and 2-(2-phenylphenoxy)ethyl methacrylate (PPEM) (Figure 2) were manufactured by Ivoclar Vivadent AG (Vaduz, Liechtenstein). Cumene hydroperoxide (CHP), acting as an oxidizing agent, was obtained from Alfa Aesar (Paris, France) (70% in water). Copper(II) acetylacetonate (Cu(acac)_2_) was obtained from Sigma Aldrich (Saint-Quentin-Fallavier, France). Thioureas were used in experiments as reducing agents. Hexanoyl thiourea (**TU1**), N,N-dibutythiourea (**TU3**), and 1-(pyridin-2-yl)thiourea (**TU6**) were provided by Ivoclar Vivadent AG. N-(n-Hexyl)thiourea (**TU2**) was obtained from abcr GmbH. N-benzoylthiourea (**TU4**) was obtained from TCI Europe. N-phenylthiourea (**TU5**) was provided from Alfa Aesar. A barium-aluminum-borosilicate glass (GM27884 d_50_ = 1.0 µm, Schott (Mainz Germany)) and a spherical SiO_2_–ZrO_2_ mixed oxide (Spherosil, Transparent Materials) were used as fillers. Spherosil is made up of agglomerates (size < 6 µm) of spherical nanoparticles (d50 = 60 nm). Before use, both fillers were modified with the silane coupling agent 3-methacryloyloxy-propyltrimethoxysilane (MPTS, Union Carbide (New York, USA)). The glass filler GM27884 and Spherosil were both silanized with 6.0 wt% MPTS.

### 2.2. Evaluation of Unfilled Materials

#### 2.2.1. Preparation of the Formulations

For the evaluation of each redox initiator system, two components were prepared. The first one contained CHP as an oxidizing agent. CHP of 1.25 wt% was added to a Bis-GMA/UDMA/PPEM: 30/40/30: wt/wt/wt monomer mixture containing 0.050 wt% of 4-methoxyphenol (MEHQ; Figure 2) as a stabilizer. The second component contained a thiourea derivative and optionally a catalytic amount of Cu(acac)_2_. In each monomer mixture, the amount of thiourea was equimolar to the molar amount of CHP to ensure a fair comparison of their effects under identical conditions. Hence, the following weight concentrations of thioureas **TUs1-6** were incorporated to a Bis-GMA/UDMA/PPEM: 30/40/30: wt/wt/wt monomer mixture containing 0.050 wt% MEHQ as a stabilizer: 1.15 wt% **TU1**, 1.05 wt% **TU2**, 1.25 wt% **TU3**, 1.20 wt% **TU4**, 1.00 wt% **TU5** and 1.00 wt% **TU6**. The different components were stirred using a SpeedMixer DAC 150.1 FVZ-K (German Engineering by Hauschild (Hamm, Germany)) at 2500 rpm for 10 min.

#### 2.2.2. Polymerization Processes Followed by Optical Pyrometry

Optical pyrometry measurements were used to follow the polymerization kinetics (Omega OS550 infrared thermometer having a sensitivity of ±1 °C for 2 g of sample). The protocol is presented in detail in [13,17,18]. The polymerization was induced by the mixing of the two components by the Sulzer Mixpac syringe (Bern, Switzerland). The sample thickness was controlled at 4 mm. The setup yielded the gel time and the maximal polymerization temperature corresponding to the maximal polymerization rate.

#### 2.2.3. Flexural Strength and Flexural Modulus

Flexural strength specimens (2 mm × 2 mm × 25 mm, n = 8) were prepared using a stainless-steel mold. A monomer mixture containing the oxidizing agent and a second mixture containing the reducing agent and the metal salt were mixed together by hand for 20 s (1/1: wt/wt). After mixing, the mold was filled with the material. The mold was then covered with a polyethylene film and was placed in an oven at 37 °C for 45 min. The specimens were removed from the mold and were stored in water at 37 °C for 24 h. The measurement of the flexural strength and modulus was carried out in three-point bending tests (span: 20 mm) with a speed of 0.8 mm min^−1^ using a Z2.5/TS universal testing machine (Zwick, (Ars-Laquenexy, France)).

### 2.3. Evaluation of Filled Materials

#### 2.3.1. Formulation of Self-Cure (SC) Composites

Composites were prepared by adding 50 wt% of a barium-aluminum-borosilicate glass filler (GM27884 d_50_ = 1.0 µm) and 10 wt% of a spherical SiO_2_–ZrO_2_ mixed oxide to the corresponding monomer mixtures. The experimental composites were mixed using a Speedmixer DAC 600.0 VAC-P (Hauschild, Hamm, Germany) at 2350 rpm for 2 min and at 2000 rpm with a vacuum of 100 mbar for 2 min. Afterwards, Sulzer Mixpac two-component syringes were filled with a composite containing the oxidizing agent CHP (**CA**) and a composite containing a thiourea and a catalytic amount of Cu(acac)_2_ (**CB**) to provide the desired SC composites (ratio **CA**/**CB**: 1/1 (vol/vol)).

#### 2.3.2. Flexural Strength and Flexural Modulus

Flexural strength specimens (2 mm × 2 mm × 25 mm, n = 8) were prepared using a stainless-steel mold. The mold was filled with the SC composite using the corresponding Sulzer Mixpac two-component syringe. The mold was then covered with a polyethylene film and was placed in an oven at 37 °C for 45 min. The specimens were removed from the mold and stored in water at 37 °C for 24 h. The measurements of the flexural strength and modulus were carried out in three-point bending tests (span: 20 mm) with a speed of 0.8 mm min^−1^ using a Z2.5/TS universal testing machine (Zwick, Ulm, Germany).

#### 2.3.3. Determination of the Double-Bond Conversion by NIR Spectrometry

Disc specimens (d = 15 mm, h = 1 mm, n = 4) were prepared with each SC composite using the corresponding Sulzer Mixpac two-component syringes. The mold was filled with the SC composite using the corresponding Sulzer Mixpac two-component syringe. After curing in an oven at 37 °C for 45 min (dry), the specimens were stored in distilled water at 37 °C for 24 h before measurement. To obtain the DBC, the spectrum of the uncured (formulation without redox initiators) and cured composite were measured by NIR spectroscopy using the Invenio-R (Bruker) spectrometer (16 scans, 8 cm^−1^ resolution, 3000–10,000 cm^−1^) at a film thickness of 1.0 mm. The methacrylate overtone peak at 6165 cm^−1^ was integrated for both spectra. DBC was calculated using the following equation:


$$DBC=(1-(\frac{A_{cured}}{A_{umcured}}))*100$$

*A_cured_* and *A_uncured_* correspond to the integrated area in the NIR spectrum of the cured and uncured composite.

#### 2.3.4. Working Time

The working time was determined with an oscillating rheometer (Rheometer MCR 302, Anton Paar, (Paris, France)) using a parallel plate geometry, at 28.7 °C. The storage modulus was recorded as a function of time (frequency = 1 Hz). A graph representing the storage modulus (logarithmic) as a function of time (nonlogarithmic) was then used for the determination of the working time. The inflexion point (to be found where the slope of the function reaches its highest value) was identified. A second point on the curve was set as soon as the storage modulus started to rise (end of the stable phase). A straight line was then drawn between the two points. The point on the curve (before the inflexion point) where the tangent line is parallel to this straight line gives the working time.

### 2.4. Evaluation of the Chemical Mechanisms of Thiourea-Based Redox Systems

#### 2.4.1. UV-Vis Experiment

UV-vis experiments were carried out using Jasco V-750 Spectrometer (les Ullis, France) in the range from 200 to 800 nm to study the absorption spectra of thioureas (**TU1** to **TU6**) in Cu(acac)_2_ solutions in tetrahydrofuran (THF).

#### 2.4.2. Electron Spin Resonance

ESR-ST experiments were performed using a X-band spectrometer (Bruker EMX plus, Ettlingen, Germany). Thioureas (**TU1** to **TU6**) were analyzed in Cu(acac)_2_ solutions in dichloromethane (DCM). The prepared formulations were studied in glass capillary tubes at room temperature under air. ESR spectra simulations were performed by WINSIM software (Houston, Texas, USA).

#### 2.4.3. Cyclic Voltammetry

The redox potentials of thioureas (**TU1** to **TU6**) were measured by cyclic voltammetry using tetrabutylammonium hexafluorophosphate as supporting electrolyte in acetonitrile (Origalys software, Lyon, France; the working electrode was a platinum disk and a saturated calomel electrode-SCE was used as the reference). All potentials were determined from half-peak potentials under nitrogen atmosphere.

#### 2.4.4. Mass Spectrometry Analysis of Initiating Fragment

High-resolution mass spectrometry experiments were performed using a Synapt G2 HDMS instrument from Waters (Manchester, UK) equipped with an electrospray ionization (ESI) source operated in the positive ion mode (capillary voltage, +2.8 kV; sampling cone voltage, +20 V; extraction cone voltage, +6 V), using a desolvation gas (N_2_) flow rate of 100 L h^−1^ at 35 °C. Ions were measured with the time-of-flight (TOF) mass analyzer, either directly from the ESI source (MS mode) or after precursor ions were selected with a quadrupole and submitted to collision with argon to obtain their product ions (MS/MS mode). The examined sample was prepared by placing the initiator system (0.15 g CHP, 0.15 g **TU6** and 0.003 g of Cu(acac)_2_) in a monomer (4 g of methyl methacrylate to generate polymethyl methacrylate (PMMA)). This sample was then dissolved in methanol, then diluted (1/10^4^, *v*/*v*) in methanol supplemented with ammonium acetate (3 mM), NaCl (0.1 mM) or LiF (0.1 mM). A syringe pump was used to introduce these sample solutions in the ESI source at a 10 μL min^−1^. Instrument control, data acquisition, and data processing of all experiments were achieved with the MassLynx 4.1 programs provided by Waters.

#### 2.4.5. Molecular Modelling

Different N-H bond dissociation energies were calculated for **TU1** and **TU6** using the Gaussian 09 Software using Density Functional Theory calculations at B3LYP/6-31G* level (Wallingford, Connecticut, USA). The spin densities on the radical centers are also determined for the generated radicals at the same level of theory.

## 3. Results and Discussion

The strategy used in this work can be presented. This study aimed to investigate the potential of new redox initiator systems based on various thiourea derivatives (**TUs1–6**) combined with CHP and a copper catalyst (Cu(acac)_2_) for the polymerization of two-component materials. First, the approach involved a systematic evaluation of filler-free formulations using pyrometry to monitor curing kinetics, followed by mechanical testing to assess flexural strength and modulus. The most promising systems were further explored by varying the catalyst concentration to optimize polymerization behavior and mechanical performance. In parallel, a detailed analysis of the underlying chemical mechanisms was carried out using cyclic voltammetry, UV-vis, ESR, and mass spectrometry to better understand the structure–reactivity relationships. Finally, selected systems showing the best balance between reactivity and mechanical properties were tested in filled composite formulations, with attention given to working time and curing efficiency, in order to assess their suitability for applications such as dental materials.

### 3.1. Evaluation of CHP//TUs1-6 + Cu(acac)_2_ Redox Initiator Systems in Filler-Free Two-Component Materials via Pyrometry

**Figure 3:**
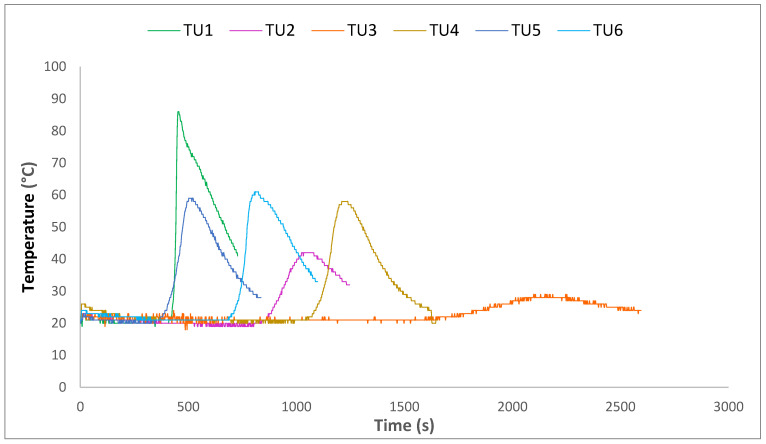
Pyrometry measurement of the investigated thioureas. System: 1.25 wt% CHP//0.01% Cu(acac)_2_ + 1.15 wt% **TU1**, 1.05 wt% **TU2**, 1.25 wt% **TU3**, 1.20 wt% **TU4**, 1.00 wt% **TU5** or 1.00 wt% **TU6**.

In order to evaluate the efficiency of redox initiator systems based on thioureas **TUs1-6**, two-component formulations were firstly prepared. The first component contained a Bis-GMA/UDMA/PPEM monomer mixture, as well as CHP as an oxidizing agent. On the other hand, the second component included the same monomer mixture combined with a thiourea derivative (equimolar amount to CHP) and a catalytic amount (100 ppm) of metal salt (Cu(acac)_2_). For each thiourea containing formulation, both components were mixed, and the curing kinetics were followed by pyrometry (Figure 3).

Interestingly, the results clearly show that the nature of the thiourea has a strong influence on the polymerization rate. The order of reactivity followed the trend: **TU1**~**TU5** > **TU6** > **TU2** > **TU4** > **TU3** with shorter gel time for the most efficient redox systems.

### 3.2. Determination of the Flexural Strength/Modulus of Filler-Free Two-Component Materials Based on CHP//TUs1-6 + Cu(acac)_2_ Redox Initiator Systems

The mechanical properties (flexural strength/modulus) of the SC two-component materials containing a thiourea and 0.01 wt% of Cu(acac)_2_ in the second component were subsequently determined (Table 1).

**Figure 4:**
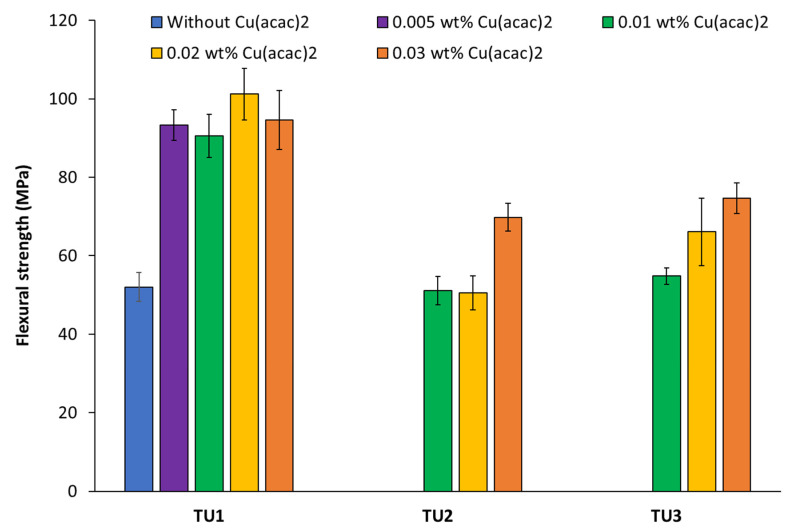
Flexural strength of SC filler-free two-component materials based on the CHP//**TU1** + Cu(acac)_2_, CHP//**TU2** + Cu(acac)_2_ and CHP//**TU3** + Cu(acac)_2_ redox initiator systems.

**Figure 5:**
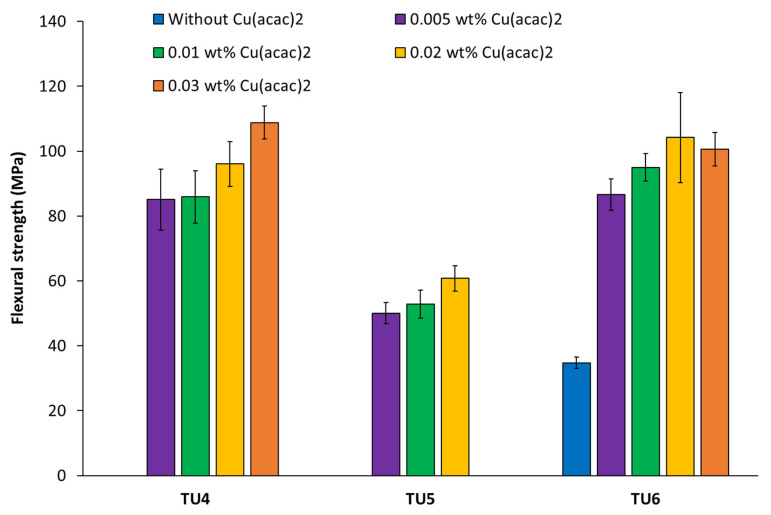
Flexural strength of SC filler-free two-component materials based on the CHP//**TU4** + Cu(acac)_2_, CHP//**TU5** + Cu(acac)_2_ and CHP//**TU6** + Cu(acac)_2_ redox initiator systems.

**Figure 6:**
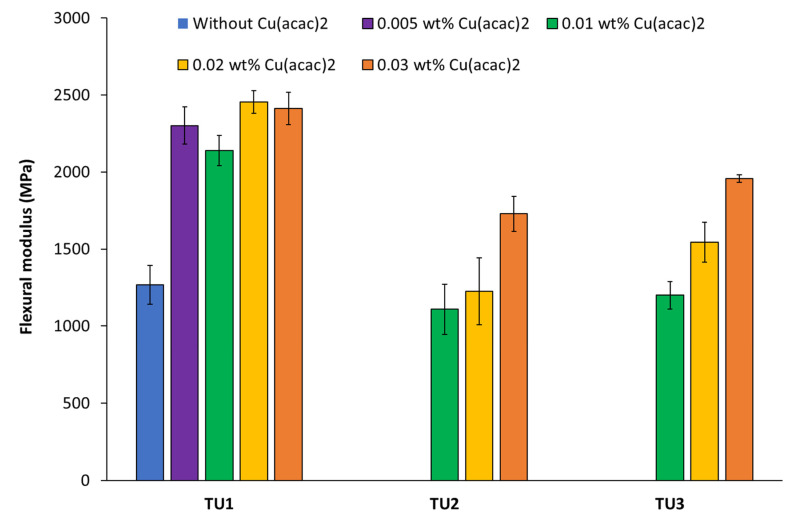
Flexural modulus of SC filler-free two-component materials based on the CHP//**TU1** + Cu(acac)_2_, CHP//**TU2** + Cu(acac)_2_ and CHP//**TU3** + Cu(acac)_2_ redox initiator systems.

**Figure 7:**
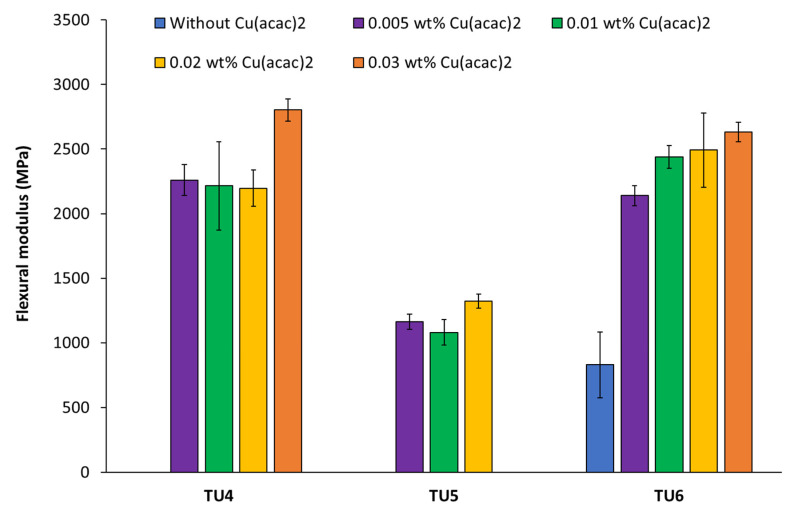
Flexural modulus of SC filler-free two-component materials based on the CHP//**TU4** + Cu(acac)_2_, CHP//**TU5** + Cu(acac)_2_ and CHP//**TU6** + Cu(acac)_2_ redox initiator systems.

The results clearly demonstrate that both the flexural strength and modulus are strongly dependent on the nature of the selected thiourea derivative. Although formulations containing **TU1**, **TU4**, or **TU6** led to excellent mechanical properties, materials based on **TU2**, **TU3**, or **TU5** provided significantly lower flexural strength and modulus values. The comparison between **TU1** and **TU2**, as well as between **TU4** and **TU5**, highlights that the presence of a carbonyl group linked to the thiourea moiety significantly improves the performance of the redox initiator systems. The higher efficiency of **TU6** in comparison to **TU5** indicates that the replacement of the phenyl group with a pyridyl moiety is a powerful strategy to reach higher flexural strength and modulus values. In order to evaluate the influence of the amount of metal catalyst on the mechanical properties of **TUs1-6**-based two-component materials, formulations containing various amounts (0.005, 0.02 and 0.03 wt%) of Cu(acac)_2_ in the second component were prepared. Metal-catalyst-free formulations were evaluated as well. The flexural strength and modulus of the corresponding SC materials were assessed. The results are presented in Figure 4, Figure 5, Figure 6 and Figure 7. In the absence of metal catalyst, formulations based on **TU2**, **TU3**, **TU4**, and **TU5** were not able to properly cure. For this reason, the mechanical properties could not be determined. The same observation could be made regarding formulations containing 0.005 wt% Cu(acac)_2_ combined with either **TU2** or **TU3**. The opposite issue was observed with **TU5**. Although moderate mechanical properties were obtained with this reducing agent, the polymerization was quite fast. Using 0.03 wt% of metal catalyst, the polymerization was so fast that the flexural strength specimens could not be properly prepared. The results also confirm that **TU1**, **TU4** and **TU6** are the most efficient reducing agents. Furthermore, they highlight the essential role of the metal catalyst for an efficient curing. Indeed, even if polymerization occurred using **TU1** and **TU6**, the resulting flexural strength/modulus of Cu(acac)_2_-free materials were significantly lower.

All materials based on the CHP/**TU1**/Cu(acac)_2_ redox initiator systems exhibited similar flexural strength/modulus values, independently of the amount of catalyst. A similar trend was observed with **TU5**. Only a slight increase in the flexural strength was observed with higher contents of metal salt. On the other hand, the Cu(acac)2 amount had a strong influence on the mechanical properties of two-component materials containing **TU2**, **TU3**, and **TU6**: the higher the amount, the higher the flexural modulus. A positive effect was also observed regarding the flexural strength. Nevertheless, even if improved mechanical properties can be obtained with **TU2** and **TU3** containing materials by adjusting the copper catalyst amount, their performance remains quite low in comparison to the **TU1**-based materials. The **TU4**-based formulations required a higher content of copper salt to reach excellent mechanical properties. Indeed, the incorporation of 0.03 wt% Cu(acac)_2_ in the second component led to the highest flexural strength and modulus values that were measured for all SC materials.

### 3.3. Investigation of the Chemical Mechanisms: Cyclic Voltammetry (CV); UV Analysis; ESR Experiments and Mass Spectrometry (MS) Analysis of Initiating Fragments

**Figure 8:**
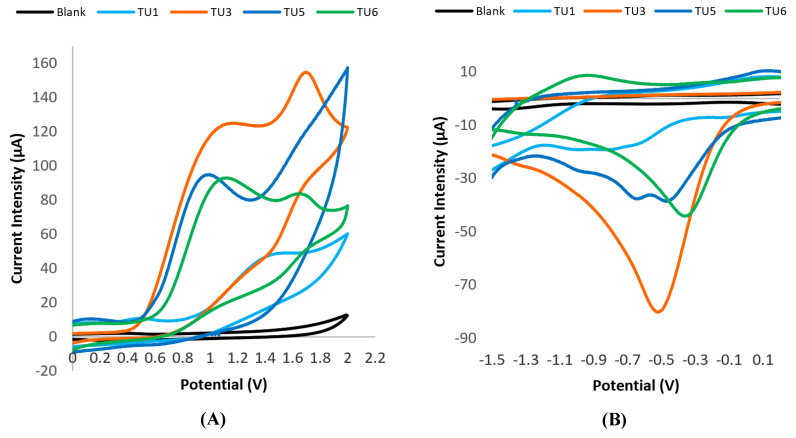
(**A**) Oxidation potentials; (**B**) reduction potential of the investigated thioureas in acetonitrile.

First, by CV experiments, the redox behavior of TUs was investigated (Figure 8); both oxidation and reduction potentials can be determined (Table 2). Interestingly, it is found that these redox potentials are affected by the structures (from −0.2 to −0.78 V for the reduction potentials (E_red_) and from +0.78 to +1.15 V for the oxidation potentials (E_ox_)). With high E_red_ and low E_ox_, it can be assumed that TUs can play the dual role of oxidizing and reducing agents. In this work, we see from ESR data below that TUs are mainly used to reduce Cu(II) in Cu(I). However, no direct relationship is found between the redox potentials and the gel time, suggesting that other factors (ligand behavior) must be taken into account.

**Figure 9:**
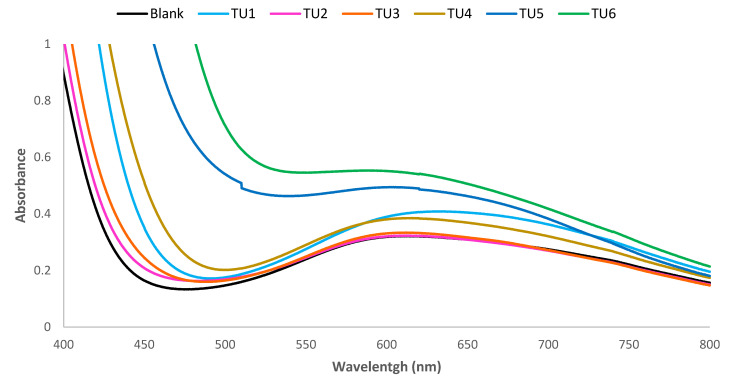
Absorbance spectra of the investigated thioureas, [TU1] = 0.0192 mol/L; [TU2] = 0.0207 mol/L, [TU3] = 0.0177 mol/L, [TU4] = 0.0185 mol/L, [TU5] = 0.0219 mol/L and [TU6] = 0.0218 mol/L in 5 × 10^−3^ Cu(acac)_2_ [blank] solution in THF.

Interestingly, in the presence of TU, the UV-vis spectrum of Cu(acac)_2_ is affected, suggesting an interaction between the metal center and TU by ligand exchange reaction of change of its oxidation degree (Figure 9, Table 3). Indeed, UV-Vis spectroscopy is a technique that is highly sensitive to both the structure and the oxidation state of such complexes [29]. To distinguish these two latter mechanisms, ESR experiments were carried out to investigate the formation of silent Cu(I) through a Cu(II) reduction by TU.

**Figure 10:**
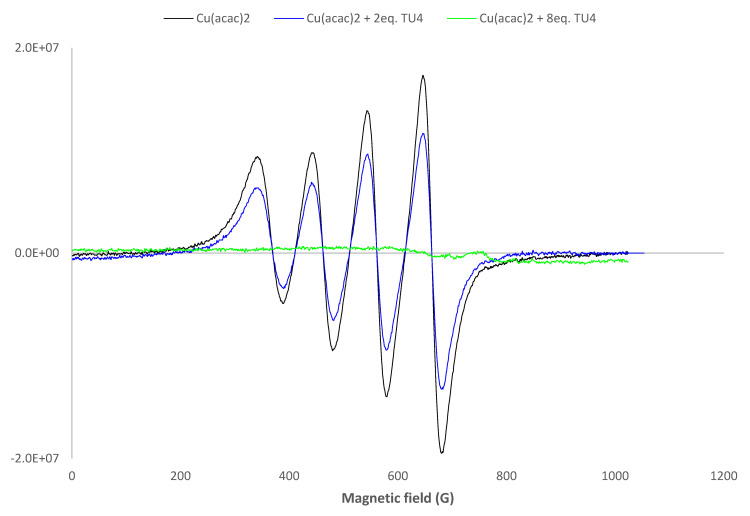
Electron spin resonance spectra of 2.18 × 10^−3^ mol/L (blue) and 8.72 × 10^−3^ mol/L (green) of **TU4** in presence of 1.09 × 10^−3^ mol/L of Cu(acac)_2_ in dichloromethane.

**Figure 11:**
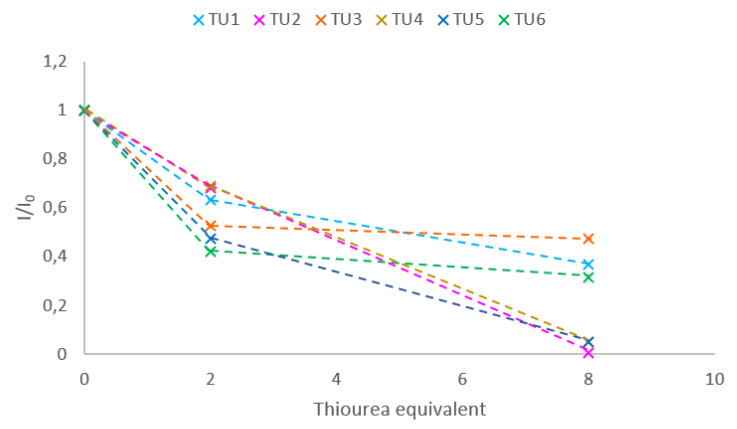
I/I0 for different thiourea equivalent vs. copper complex concentration. I and I0 correspond to the double integration of the ESR spectra of copper complex with thiourea (I) and without thiourea (I0), respectively.

Markedly, in the presence of TU, the ESR signal ascribed to Cu(II) strongly decreased (Figure 10 and Figure 11), clearly suggesting a Cu(II) reduction in Cu(I) in agreement with the reductive behavior of TU (see CV data). The stoichiometry between Cu and TU affects the reduction process, i.e., more TU leading to a huge decrease of the Cu(II) form. This can be associated with a ligand exchange followed by reduction process.

**Figure 12:**
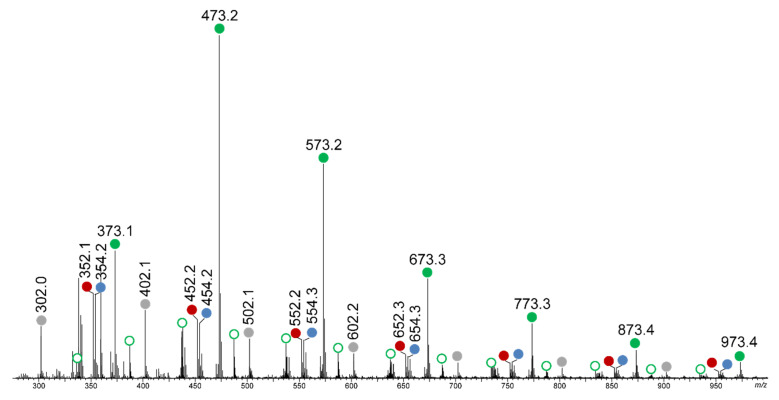
Positive-mode ESI-MS of the sample (in methanol supplemented with ammonium acetate) obtained by mixing CHP + Cu(acac)_2_ + **TU6** (initiating system) with MMA.

**Figure 13:**
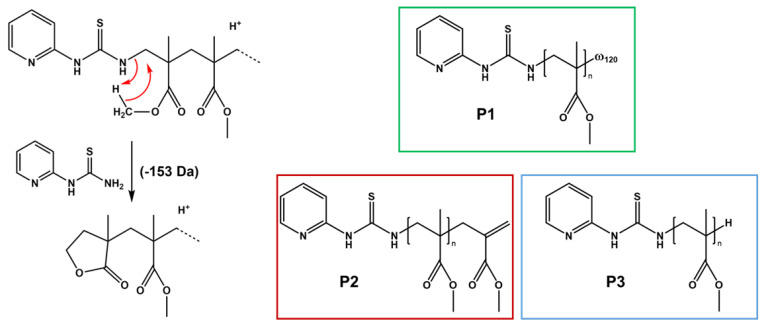
(**Left**) Elimination of a 153 Da neutral during MS/MS of **TU6**-initiated PMMA. (**Right**) Structure proposed for the three main PMMA detected in ESI-MS.

To determine more in detail the initiating structures in this system, mass spectrometry experiments were performed for end-group analysis of PMMA obtained via the redox polymerization of MMA using CHP/**TU6**/Cu(acac)_2_ as the initiating system. Using positive-mode electrospray ionization (ESI), data recorded in the MS mode (Figure 12) showed four main PMMA distributions characterized by peaks spaced by 100 Da when singly charged (filled circles) or 50 Da when doubly charged (empty circles). Using different salts yielded the ionic species observed in Figure 12 when using ammonium acetate, which were determined to be protonated molecules (except ions designated with grey symbols that did not experience any *m*/*z* shift when changing the cationization agent). When submitting protonated oligomers to collision-induced dissociation, elimination of a 153.0 neutral (C_6_H_7_N_3_S, according to accurate mass measurements) was found to occur from the three polymers named P1 (in green), P2 (in red), and P3 (in blue), allowing their α group to be assigned to **TU6**_(-H)_. The chain end corresponds to **TU6** that has lost a hydrogen atom. MS does not allow us to determine which hydrogen has been abstracted. The structures represented for P1, P2, and P3 (Figure 13) correspond to abstraction on the NH_2_ group, but N-H abstraction is also possible. While the 120 Da ω termination of P1 could not be determined, accurate mass measurements permitted to validate the structures proposed for P2 and P3 (Figure 13). All these data clearly highlight the initiating role of the TU being the initiating fragment.

**Scheme 1:**
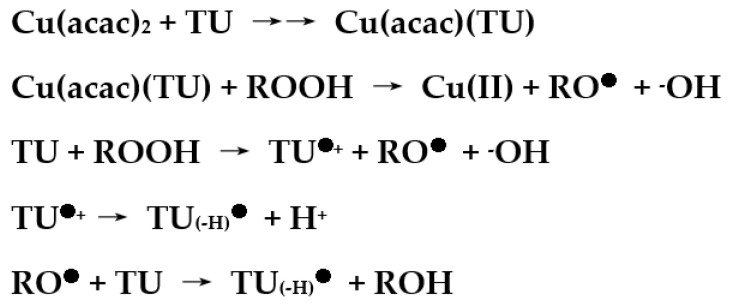
Proposed chemical mechanisms (with ROOH = CHP).

Based on these results, some chemical mechanisms can be proposed. Only a part of the chemical mechanisms is given in Scheme 1 (formation of a new Cu(I) complex with TU as shown in ESR and UV-vis spectroscopy experiments) as other side reactions affecting the structure/reactivity relationship are expected. **TU_(-H)_^●^** can be considered as the main initiating fragment as shown by MS polymer analysis.

Some molecular modeling calculations were carried out at B3LYP/6-31G* level on potential structures for **TU_(-H)_^●^** depending on the hydrogen abstraction position (Table 4). Interestingly, the N-H abstraction position is more favorable than the NH_2_ position (lower N-H BDE) and a significant participation of the sulfur atom is found. Markedly, the structure of the potential radicals derived from **TU1** and **TU6** are very different (the spin densities being very different, i.e., the radical localization is strongly influenced by the TU structure); this suggests very different initiating ability from the different TUs.

The understanding of the structure–chemistry/reactivity/efficiency relationship remains at the core of chemists’ concerns in order to develop new systems. However, in the redox system CHP/copper complex + thiourea, this understanding is quite complex because many factors influence the reactivity of thioureas. The list of factors that can affect their reactivity includes the following: (i) their redox properties (allowing them to act as a reducing agent, e.g., for Cu(II) and CHP); (ii) the ability of thiourea to act as a ligand in copper complexes (i.e., its aptitude for ligand exchange); (iii) the reactivity of the formed radicals (as they initiate according to polymer chain-end analysis results in MS). From this study, it appears that reactivity is not governed by a single factor but rather results from a combination of several factors, preventing the establishment of a direct structure–reactivity relationship. In principle, the formation of TU_(-H)_**^●^** radicals can occur without the involvement of Cu(II). However, our experimental results clearly demonstrate that the presence of Cu(acac)_2_ is essential for the reaction to proceed efficiently. In its absence, radical formation is significantly less effective, confirming that Cu(II) plays a key catalytic role in enhancing the reactivity of the thioureas.

### 3.4. Evaluation of Two-Component Flowable Composites Based on CHP//TU1, TU4, or TU6 + Cu(acac)2 Redox Initiator Systems

The most promising redox initiator systems (containing **TU1**, **TU4**, or **TU6** as a reducing agent) were subsequently evaluated for the curing of two-component flowable composites. Hence, 50.0 wt% of glass filler GM27884 (1.0 µm) and 10.0 wt% mixed oxide Spherosil were added to both components of the filler-free formulations previously evaluated (Section 3.2). For each material, the mechanical properties (flexural strength/modulus), the working time and the DBC were determined (Table 5). Similarly to the results presented with filler-free formulations, all **TU1**-based composites led to similar flexural strength/modulus values, regardless of the Cu(acac)_2_ amount. Moreover, measured DBCs were not significantly different. As expected, the amount of copper catalyst strongly influenced the working time: The higher the Cu(acac)_2_ content, the faster the polymerization. A working time of roughly 120–180 s is typically well suited for two-component flowable dental composites. Significantly longer working times are not desired, as the dentist would have to wait too long before a proper curing occurs. On the other hand, significantly shorter working times are also problematic, as the dentist needs time to properly fill the cavity before curing. In this context, the material based on the CHP//**TU1** + 0.01 wt% Cu(acac)_2_ seems ideal (working time = 132 ± 1 s). The two other tested materials cured either too fast (CHP//**TU1** + 0.02 wt% Cu(acac)_2_; working time = 88 ± 3 s) or too slow (CHP//**TU1** + 0.005 wt% Cu(acac)_2_; working time = 242 ± 6 s). Composites containing the CHP//**TU4** + Cu(acac)_2_ redox initiator system led to the highest flexural strength and modulus values. These composites polymerized significantly slower than the **TU1**-based materials. Therefore, it can be claimed that the conjugation of the carbonyl group with a phenyl ring significantly reduces the reactivity of the corresponding acyl thiourea. The amount of copper catalyst was further increased to improve the working time. The addition of 0.05 wt% Cu(acac)_2_ in the monomer mixture of the second component was necessary to reach a working time (193 ± 3 s) that was close to the optimal range. The CHP//**TU6** + Cu(acac)_2_ redox initiator system provided the fastest polymerization. Hence, the results showed that, contrary to the phenyl ring, the conjugation of the carbonyl with a pyridyl group leads to an acceleration of the redox polymerization. This result was unexpected, as **TU1** was shown to be more reactive than **TU6** in the pyrometry study. It seems, therefore, that the presence of the inorganic fillers plays a significant role in the reactivity of the redox initiator systems. The strong difference of viscosity or an interaction between the fillers and the TU might explain this observation. The **TU6**-based composites containing the smallest amounts of copper salt (0.005 and 0.01 wt%) led to suitable working times (169 ± 8 s and 115 ± 3 s, respectively), whereas the remaining formulation (0.02 wt% Cu(acac)_2_) polymerized too fast (81 ± 2 s). All three materials provided similar DBCs than the composites based on **TU1** or **TU4**. As a whole, the comparison of the results obtained with **TU1**, **TU4**, and **TU6** shows that each of these thiourea derivatives can be successfully used as a reducing agent in two-component flowable composites, as long as the amount of copper catalyst has been properly selected.

## 4. Conclusions

In summary, this study demonstrates that the structure of thiourea compounds plays a decisive role in the efficiency of redox polymerization systems. Through a combination of experimental techniques and molecular modeling, we showed that specific structural modifications—particularly the incorporation of carbonyl or pyridyl groups—significantly enhance the reactivity of the thiourea moiety, enabling rapid initiation, and excellent polymerization kinetics. Among the six thioureas evaluated, **TU1**, **TU4**, and **TU6** stood out by producing self-curing materials with high flexural strength and modulus. These three thioureas were further integrated into two-component dental flowable composites, which exhibited a favorable balance of mechanical performance and practical working time, provided that the Cu(acac)_2_ concentration was appropriately adjusted.

Overall, this work not only elucidates key structure–reactivity relationships in thiourea-based redox systems but also demonstrates their applicability in high-performance, room-temperature polymer formulations. These findings pave the way for the design of next-generation redox polymerization strategies for dental materials and other advanced applications, such as surgical adhesives and sealants, in line with the growing demand for sustainable, efficient, and ambient-curing technologies.

## Figures and Tables

**Table 1:** Flexural strength and flexural modulus of SC two-component materials based on the CHP//**TUs1-6** + Cu(acac)_2_ redox initiator system.

Oxidizing Agent/Reducing Agent	Cu(acac)_2_ (wt% in the Second Component)	Flexural Strength (MPa)	Flexural Modulus (MPa)
**CHP//TU1**	0.01	90.5 ± 5.5	2065 ± 154
**CHP//TU2**	0.01	51.1 ± 3.6	1108 ± 163
**CHP//TU3**	0.01	54.8 ± 2.1	1200 ± 89
**CHP//TU4**	0.01	85.9 ± 8.1	2216 ± 342
**CHP//TU5**	0.01	52.9 ± 4.3	1080 ± 99
**CHP//TU6**	0.01	95.0 ± 4.2	2437 ± 88

**Table 2:** Redox potentials of the investigated thioureas in acetonitrile (vs. SCE).

Systems	E_ox_ (V)	E_red_ (V)
**TU1**	1.15	−0.55
**TU2**	0.85	−0.35
**TU3**	0.78	−0.33
**TU4**	1.15	−0.78
**TU5**	0.79	−0.35
**TU6**	0.80	−0.20

**Table 3:** Color of the investigated thioureas in 5 × 10^−3^ Cu(acac)_2_ solution in THF.

Thiourea	TU1	TU2	TU3	TU4	TU5	TU6
[TU] (mol/L)	0.0192	0.0207	0.0177	0.0185	0.0219	0.0218
Color	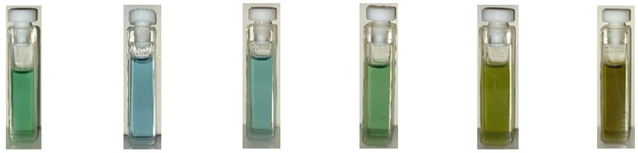					

**Table 4:** DFT parameters (N-H Bond Dissociation Energies BDE, and spin densities on the Sulfur and Nitrogen atoms for the generated radical).

Position of the H-Abstraction	BDE N-H (kcal/mol)	Spin Density (N)	Spin Density (S)
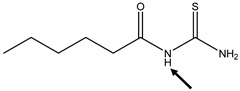	81.1	0.02	0.71
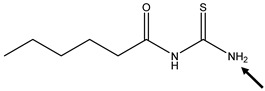	93.5	0.00	0.86
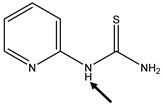	96.3	0.5	0.34
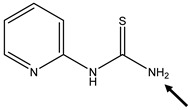	104.9	0.39	0.7

**Table 5:** Flexural strength, flexural modulus, working time, and DBC of SC two-component flowable composites based on the CHP//**TU1** + Cu(acac)_2_, CHP//**TU4** + Cu(acac)_2,_ and CHP//**TU6** + Cu(acac)_2_ redox initiator systems.

Oxidizing Agent/Reducing Agent	Cu(acac)_2_ (wt% in the Monomer Mixture)	FS (MPa)	FM (MPa)	Working Time (s)	DBC (%)
**CHP//TU1**	0.005	113.4 ± 9.9	6321 ± 237	242 ± 6	79 ± 1
**CHP//TU1**	0.01	107.9 ± 4.7	6241 ± 263	132 ± 1	77 ± 1
**CHP//TU1**	0.02	107.4 ± 9.9	6419 ± 246	88 ± 3	77 ± 2
**CHP//TU4**	0.01	122.0 ± 10.5	7132 ± 213	440 ± 31	76 ± 1
**CHP//TU4**	0.02	117.8 ± 8.5	7212 ± 127	280 ± 14	77 ± 1
**CHP//TU4**	0.03	114.6 ± 8.8	6856 ± 103	236 ± 29	78 ± 2
**CHP//TU4**	0.05	117.4 ± 8.4	7041 ± 251	193 ± 3	78 ± 1
**CHP//TU6**	0.005	120.9 ± 8.2	6219 ± 221	169 ± 8	81 ± 1
**CHP//TU6**	0.01	104.6 ± 11.0	6710 ± 317	115 ± 3	79 ± 3
**CHP//TU6**	0.02	115.7 ± 6.9	6788 ± 355	81 ± 2	80 ± 1

## Data Availability

The data that support the findings of this study are available from the corresponding authors, upon reasonable request.
